# Red and processed meat and risk of colorectal cancer: an update

**DOI:** 10.17179/excli2018-1554

**Published:** 2018-08-08

**Authors:** Bachir Benarba

**Affiliations:** 1 Laboratory Research on Biological Systems and Geomatics, Faculty of Nature and Life,University of Mascara, Algeria

## ⁯

Dear Editor,

Colorectal cancer is the third most common malignancy worldwide and the fourth most leading cause of cancer deaths. Colorectal cancer has become a real public health issue in both developed and developing countries (Ansa et al., 2018[4]; Favoriti et al., 2016[12]). Several risk factors are associated with colorectal cancer such as obesity, smoking, alcohol, advanced age and high intake of red and processed meat (Nasrallah and El-Sibai, 2014[19]). High incidence of colorectal cancer risk factors including red and processed meat has been reported in different countries such as Jordan (Tayyem et al., 2017[30]), Slovakia (Spáčilová et al., 2018[27]), and China (Gu et al., 2018[14]).

Numerous studies have demonstrated that high intake of red and processed meat could be linked to an increased risk of colorectal cancer (Bernstein et al., 2015[5]; Oostindjer et al., 2014[20]). It seems that red meat may activate Toll-like receptors at the intestinal epithelial surface and triggers the NF-κB inflammatory pathway, resulting in colorectal cancer (Kopp et al., 2018[15]). In addition, carcinogenic compounds such as heterocyclic aromatic amines, N-nitroso-compounds, and polycyclic aromatic hydrocarbons are produced when meat is cooked at high temperatures (Domingo and Nadal, 2017[10]).

High intake of red and processed meat has been also linked to an increased risk of several cancers. Recently, Rosato et al. (2017[22]) analyzed all the case-control studies carried out in Italy from 1982 to 2006. They found that high intake of processed meat (≥ 20 g/day) was associated with increased risk of breast, ovarian and endometrial cancers. Similar findings were reported in the UK (Anderson et al., 2018[2]). In a prospective study, Diallo et al. (2018[9]) found that high intake of red and processed meat was associated with a higher risk of overall cancers and breast cancer. In addition, increased intake of red and processed meat was found to be positively linked to a higher risk of chronic obstructive pulmonary disease (Salari-Moghaddam et al., 2018[24]).

In the present letter, we summarize the recent studies carried out to investigate the association between red and processed meat intake and colorectal cancer risk (Table 1(Tab. 1); References in Table 1: Gigic et al., 2018[13]; Shin et al., 2018[26]; Gu et al., 2018[14]; Wada et al., 2017[34]; Wei et al., 2017[35]; Carr et al., 2017[7]; Torres Stone et al., 2017[31]; Rosato et al., 2017[23]; Vieira et al., 2017[32]; Farchi et al., 2017[11]; Vulcan et al., 2017[33]; Lippi et al., 2016[16]; Zhao et al., 2017[36]; Angelo et al., 2016[3]; Aithal et al., 2017[1]; Tamakoshi et al., 2017[29]; Brenner et al., 2017[6]; Lourenço et al., 2018[17]; Rada-Fernandez de Jauregui et al., 2018[21]; Tabung et al., 2017[28]; Schwingshackl et al., 2018[25]; De Vries et al., 2017[8]; Mehta et al., 2017[18]).

| **Type of study/ participants** | **Country** | **Key findings** | **Reference** |
|---|---|---|---|
| Retrospective study/ 192 colorectal cancer patients | Germany | A dietary pattern characterized by high intake of red and processed meat was inversely correlated with quality of life in colorectal cancer patients, after surgery. | Gigic et al. (2018) |
| Population-based prospective cohort study | Japan | The association between dietary patterns and colorectal cancer risk was studied. Among men, the prudent pattern based on vegetables and fruits resulted in a decreased risk of both colorectal, colon and distal cancers. On the other hand, the westernized pattern featured by high intake of red and processed meat was associated with an increased risk of colon and distal cancers among women. | Shin et al. (2018) |
| Retrospective study | China | High red and processed meat intake consumption accounted for 8.6 % of colorectal cancer incidence and mortality in China in 2012. | Gu et al. (2018) |
| Population-based prospective cohort/ 13 957 men and 16 374 women | Japan | High intake of red and processed meat resulted in a significant increase of colorectal cancer risk in men, but not in women. | Wada et al. (2017) |
| Competing risks regression and statistical tests/ 1718 women from the Nurses' Health Study | USA | Increased consumption of processed meat appeared to be a risk factor for distal colon cancer. No significant correlation was found between processed meat intake and proximal colon cancer. | Wei et al. (2017) |
| Population-based case–control/ 2449 cases and 2479 controls | Germany | Increased risk of colorectal, colon and rectal cancers was associated with high intake of red and processed meat (> 1 time/day vs ≤ 1 time/week OR 1.66). This association was confirmed using molecular pathologic features of colorectal cancer: microsatellite instability, CpG island methylator phenotype, BRAF, estrogen receptor-β and p53 status. | Carr et al. (2017) |
| Prospective longitudinal study/ 398,458 persons | USA | Persons with higher intake of red and processed meat developed more colorectal cancers. | Torres Stone et al. (2017) |
| Case-control study/ 3745 cases and 6804 controls | Italy | Each increased intake of processed meat by 10 g/day resulted in an OR of 1.02 for proximal colon cancer. Furthermore, this positive association between processed meat and proximal colon cancer was stronger when the intake was of > 25 g/day for men, > 21.5 for women. | Rosato et al. (2017b) |

EXCLI Journal 2018;17:792-797 – ISSN 1611-2156
Received: July 22, 2018, accepted: August 06, 2018, published: August 08, 2018

| Type of study/ participants | Country | Key findings | Reference |
|---|---|---|---|
| Prospective studies/ 6682 cases | World | High intake of red and processed meat increases the risk of colorectal cancer. Each increase of 100 g/day of red and processed meat results in an increase in the risk of colorectal by 12 %. | Vieira et al. (2017) |
| Prospective study | Italy | A reduction of 40 % and 80 % in the beef consumption may avoid 2.3 and 4.5 % of deaths among colorectal cancer patients, respectively. | Farchi et al. (2017) |
| Retrospective study | Sweden | Increased risk of rectal cancer was found in men with high intake of beef. Likewise, higher intake of pork and processed meat was associated with enhanced risk of colorectal cancer. On the other hand, fish consumption was negatively correlated with risk of rectal cancer. | Vulcan et al. (2017) |
| Meta-analysis | World | Enhanced intake of red and processed meat was associated with increased risk of colorectal cancer. On the other hand, white meat consumption was negatively correlated with colorectal cancer. | Lippi et al. (2016) |
| Meta-analysis | World | Increased intake of red and processed meat was positively associated with the risk of overall colorectal cancer.<br>No significant association was found for rectal cancer. | Zhao et al. (2017) |
| Case-control study/ 169 cases and 101 controls | Brazil | High intake of beef, chicken and pork was associated with higher risk of sporadic colorectal cancer. | Angelo et al. (2016) |
| Case-control study/ 100 cases and 200 controls | India | Frequent consumption of red meat resulted in 1.5 folds higher risk to develop colorectal cancer. | Aithal et al. (2017) |
| Retrospective study/ 3699 men and 2165 women | Japan | Patients with rectal cancer were found to eat more meat when compared to colon cancer patients. | Tamakoshi et al. (2017) |
| Cohort study/ ND | Canada | Indirect relationship between increased incidence of colorectal cancer and enhanced intake of red and processed meat among younger adults. Sodium and preservatives added to processed meat may be responsible for this correlation. | Brenner et al. (2017) |
| Mathematic model (Prevent model) | Denmark | 19 % of colorectal cancer cases were avoidable by excluding both red and processed meat. Moreover, a reduction of 25 g/day in red and processed meat intake among men could avoid 9.1 % of colorectal cancer cases. | Lourenço et al. (2018) |

| Type of study/ participants | Country | Key findings | Reference |
|---|---|---|---|
| Multivariable-adjusted models – Analysis from a cohort study | UK | Compared to diets including red meat, red meat free diets were associated with a re-duced risk of distal colon cancer. | Rada-Fernandez de Jauregui et al. (2018) |
| Meta-analysis | World | Higher colorectal cancer risk was associated with an “unhealthy” pattern featured by high con-sumption of both red and processed meat. Lower intake of red and processed meat was considered protective against colo-rectal cancer. | Tabung et al. (2017) |
| Meta-analysis | World | Lower intake of red and processed meat was negatively correlated with colorectal cancer risk. | Schwingshackl et al. (2018) |
| Macro-simulation study | Columbia | Eliminating red and processed meat from diet resulted in a reduction of 13.5 % of colorectal cancer cases. | De Vries et al. (2017) |
| Data analy-sis | USA | High intake of red and processed meat charac-terizing a “Western” dietary pattern was associ-ated with increased risk of colo-rectal cancer in both men and women. | Mehta et al. (2017) |

## *Conflict of interest*

The author declares no conflict of interest.

## Conflict of interest

The author declares no conflict of interest.

## Figures and Tables

**Table 1:**
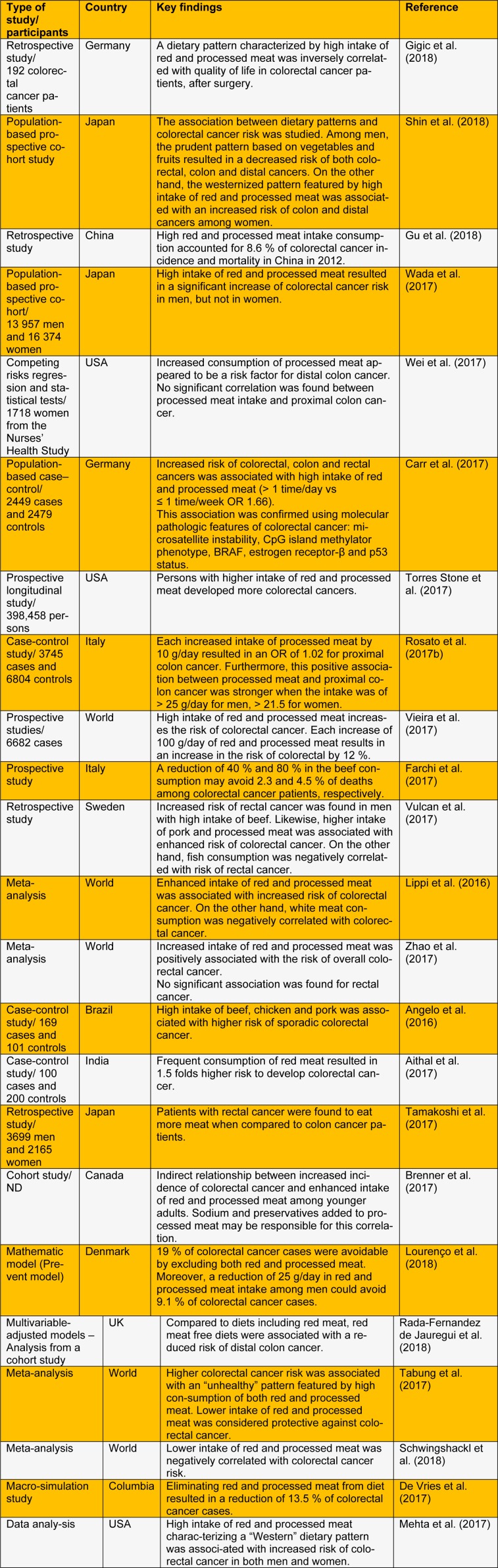
Recent studies of the association between red and processed meat-colorectal cancer risk
